# Editorial: Chronobiology in cardiometabolic health and disease

**DOI:** 10.3389/fphar.2024.1544963

**Published:** 2025-01-07

**Authors:** Cristina Manuela Dragoi, Zhihong Yang, Baharan Fekry, Andrea Brenna

**Affiliations:** ^1^ Department of Biochemistry, Faculty of Pharmacy, Carol Davila University of Medicine and Pharmacy, Bucharest, Romania; ^2^ Department of Endocrinology, Metabolism, and Cardiovascular System, Faculty of Science and Medicine, University of Fribourg, Fribourg, Switzerland; ^3^ Institute of Molecular Medicine, University of Texas Health Science Center at Houston, Houston, TX, United States

**Keywords:** chronobiology, circadian clocks, metabolic health, time restricting feeding, dietary and lifestyle interventions, molecular clocks

## General introduction and relevance of the circadian rhythms in physio-pathology

Circadian rhythms are intrinsic timekeeping systems that orchestrate the physiological processes of living organisms in alignment with the 24-h day-night cycle. These rhythms, governed by molecular clocks observed in almost every cell, regulate essential biological functions, including metabolism, hormone release, and sleep-wake cycles. Disruptions to these finely tuned systems—due to modern lifestyle, shift work, or excessive exposure to artificial light—can misalign the internal clock with environmental cues. This misalignment has been increasingly associated with a spectrum of diseases, from cardiovascular disorders and metabolic syndrome to neuropsychiatric and oncological conditions (de Assis LVM and Kramer A. Genes Dev. 2024 November 27; 38(21–24):933–951. doi: 10.1101/gad.352180.124). Understanding circadian rhythms provides an unique opportunity to explore their role as predictive and therapeutic tools for improving human health.

### Description of cardiometabolic health and disease

Cardiometabolic health, a term covering the interconnected states of cardiovascular and metabolic health, serves as a critical determinant of overall wellbeing. Cardiometabolic diseases, including diabetes, hypertension, atherosclerosis, and related heart failure, have reached epidemic proportions globally, driven by behaviors, unhealthy diet, and environmental stressors. The circadian field offers a promising lens to explore the complex etiology of these diseases (Gentile F, et al., Heart Fail Rev. 2024 October 11. doi: 10.1007/s10741-024-10447-1). Evidence suggests that disruptions in circadian rhythms can impair metabolic and cardiovascular function, highlighting their potential role in disease prediction and prevention. Moreover, circadian biology paves the way for advancements in personalized medicine, offering strategies to tailor interventions such as pharmacotherapy and lifestyle modifications to align with an individual’s chronotype, enhancing therapeutic efficacy.

## Prevention of cardiovascular and metabolic diseases based on circadian rhythms

Harnessing the circadian clock to develop preventive strategies for cardiovascular and metabolic diseases represents a paradigm shift in public health and clinical practice. Regular physical activity, timed to align with the body’s natural rhythms, has been shown to significantly improve metabolic outcomes and cardiovascular resilience. Similarly, structured meal timing, such as time-restricted eating, optimizes metabolic efficiency by synchronizing nutrient intake with the body’s circadian-driven processes. Maintaining consistent sleep-wake cycles and minimizing exposure to light during nighttime have been shown to regulate hormonal balance, reducing the risk of cardiometabolic disturbances. Additionally, pharmacological targeting of the circadian clock presents a promising strategy for restoring rhythmic balance and enhancing health outcomes. These preventive mechanisms emphasize the potential of circadian rhythm alignment as a non-invasive, cost-effective approach to improve population health.

This Research Topic, Chronobiology in Cardiometabolic Health and Disease, brings together diverse perspectives on the profound correlation between circadian rhythms and cardiometabolic health. Disruptions to the body’s internal clock are increasingly recognized as pivotal contributors to the development of cardiovascular and metabolic disorders, and this Research Topic of studies highlights promising avenues for intervention and understanding.

One focal area is the influence of exercise on circadian rhythms, showcasing its role as a non-photic zeitgeber. Shen and colleagues reviewed the effects of exercise on regulation of internal clock, which could act as a non-drug intervention for circadian rhythm disorders, with implications for improving cardiometabolic outcomes and overall health (Shen et al.).

Another theme, discussed by Chen and colleagues, is the intricate dialogue between mitochondria and the endoplasmic reticulum, uncovering their role in cellular processes such as calcium signaling, lipid transport, and autophagy. Dysregulation of these pathways is linked to aging-related cardiovascular diseases, and targeting these interactions offers a novel therapeutic approach to mitigate the effects of aging on the cardiovascular system (Chen et al.).

Ma and colleagues provided further insights into the overlapping roles of endoplasmic reticulum stress and autophagy in aging and cardiovascular diseases. This research highlights potential strategies to counteract aging-associated pathologies and protect cardiovascular health by elucidating the molecular pathways underlying these processes (Ma et al.).

The Research Topic also delves into the impact of time-restricted eating (TRE) as a promising dietary intervention. By aligning food intake with the body’s circadian rhythms, TRE has shown potential to enhance metabolic balance, particularly in individuals with obesity and metabolic disorders. A review by Ribas-Latre and colleagues emphasizes the interaction between TRE, the circadian clock, and the gut microbiota, shedding light on how these factors collectively influence cardiometabolic health. Also, evidence suggests that TRE enhances insulin sensitivity and lowers inflammatory markers, mitigating metabolic disturbances. Aligning TRE with individual chronotypes may further optimize its cardiometabolic benefits (Ribas Latre et al.).

Lastly, Özata and colleagues provided genetic insights into circadian regulation through CLOCK gene polymorphisms. This work examines how genetic variations impact circadian behaviors, dietary patterns, and metabolic health, underscoring the importance of personalized approaches to nutrition and lifestyle interventions. The results suggest that CLOCK gene variants partially modulate the relationship between chrononutrition behaviors and BMI, emphasizing the potential role of personalized nutrition based on gene-diet interactions (Uyar et al.).

Together, these studies underscore the critical role of circadian biology in maintaining cardiometabolic health and highlight emerging opportunities for therapeutic innovation. By addressing the molecular, behavioural, and genetic dimensions of chronobiology, this Research Topic aims to inspire further research and application in the fields of chronotherapy and personalized medicine.

## Conclusion

This Research Topic emphasizes the profound interplay between circadian biology and cardiometabolic health, illustrating the necessity of integrating circadian principles into preventive, diagnostic, and therapeutic strategies. By exploring circadian rhythms’ molecular, behavioural, and clinical dimensions, this Research Topic aims to inspire innovation in chronomedicine and foster a deeper understanding of how aligning daily behaviours with the body’s natural clock can revolutionize healthcare outcomes.

